# European Society of Emergency Radiology: guideline on radiological polytrauma imaging and service (short version)

**DOI:** 10.1186/s13244-020-00947-7

**Published:** 2020-12-10

**Authors:** Stefan Wirth, Julian Hebebrand, Raffaella Basilico, Ferco H. Berger, Ana Blanco, Cem Calli, Maureen Dumba, Ulrich Linsenmaier, Fabian Mück, Konraad H. Nieboer, Mariano Scaglione, Marc-André Weber, Elizabeth Dick

**Affiliations:** 1 European Society of Emergency Radiology, ESER Office, Am Gestade 1, 1010 Vienna, Austria; 2 Department of Radiology, LMU University Hospital, Munich, Germany; 3 Department of Radiology and Nuclear Medicine, Schwarzwald-Baar-Hospital, Villingen-Schwenningen, Germany; 4 Department of Neurosciences, Imaging and Clinical Science, University of Chieti, Chieti, Italy; 5 Department of Medical Imaging, Sunnybrook Health Sciences Centre, Toronto, ON Canada; 6 Department of Radiology, University Hospital JM Morales Meseguer, Murcia, Spain; 7 Department of Radiology, Ege University Medical Faculty, Izmir, Turkey; 8 Imperial College NHS Trust, St Mary’s Campus, London, UK; 9 Department of Diagnostic and Interventional Radiology, Helios Clinic Munich West, Munich, Germany; 10 Department of Radiology, University Ziekenhuis, Vrije University (VUB), Brussels, Belgium; 11 James Cook University Hospital, Teesside University, Middlesbrough, UK; 12 Department of Imaging, Pineta Grande Hospital, Castel Volturno, Italy; 13 Institute of Diagnostic and Interventional Radiology, Pediatric Radiology and Neuroradiology, University Medical Center, Rostock, Germany

**Keywords:** Europe, Guideline, Radiology, Polytrauma, Whole-body-CT

## Abstract

**Background:**

Although some national recommendations for the role of radiology in a polytrauma service exist, there are no European guidelines to date. Additionally, for many interdisciplinary guidelines, radiology tends to be under-represented. These factors motivated the European Society of Emergency Radiology (ESER) to develop radiologically-centred polytrauma guidelines.

**Results:**

Evidence-based decisions were made on 68 individual aspects of polytrauma imaging at two ESER consensus conferences. For severely injured patients, whole-body CT (WBCT) has been shown to significantly reduce mortality when compared to targeted, selective CT. However, this advantage must be balanced against the radiation risk of performing more WBCTs, especially in less severely injured patients. For this reason, we recommend a second lower dose WBCT protocol as an alternative in certain clinical scenarios. The ESER Guideline on Radiological Polytrauma Imaging and Service is published in two versions: a full version (download from the ESER homepage, https://www.eser-society.org) and a short version also covering all recommendations (this article).

**Conclusions:**

Once a patient has been accurately classified as polytrauma, each institution should be able to choose from at least two WBCT protocols. One protocol should be optimised regarding time and precision, and is already used by most institutions (variant A). The second protocol should be dose reduced and used for clinically stable and oriented patients who nonetheless require a CT because the history suggests possible serious injury (variant B). Reading, interpretation and communication of the report should be structured clinically following the ABCDE format, i.e. diagnose first what kills first.

## Key points

- If indicated, whole-body-CT (WBCT) saves lives in severely injured patients.
- However, WBCT radiation dose risk versus benefit depends on severity of injury.
- Two WBCT protocols should be established (A: time/precision optimised, B: dose reduced).
- Protocol A should be used for clinically unstable patients/life-threatening conditions.
- For all other patients, protocol B should be selected.

## Background

The European Society of Emergency Radiology (ESER) is an apolitical, non-profit organisation, exclusively and directly dedicated to promoting and coordinating the scientific, philanthropic, intellectual and professional activities of Emergency Radiology. The Society’s mission at all times is to serve the health care needs of the general public through the support of science, teaching, research and the quality of service in the field of Emergency Radiology [1]. One particular aim of ESER is to advance and improve the radiological aspects of emergent patient care and to advance the quality of diagnosis and treatment of acutely ill or injured patients using imaging.

Emergency Radiology encompasses medical and surgical subspecialties including polytrauma services. Concerning the latter, past and present ESER board members had taken part in several interdisciplinary guideline processes. However, the ESER board has observed the lack of dedicated separate independent radiological recommendations for a radiological polytrauma service. The ESER has therefore created such recommendations with the hope that this will start to bring corresponding diverse national and international radiological societies together in order to refine the statements, gain visibility for national societies and in particular, strengthen the role of radiology in upcoming interdisciplinary polytrauma guideline development.

As ESER also wants to be a promotor of future scientific work, we hope to give advice on specific questions as well as for a more general principal direction. To update this guideline, ESER will refine the statements at appropriate time intervals, (currently estimated to be two years).

The Guideline on Radiological Polytrauma Imaging and Service is published simultaneously in a comprehensive short version (this article) and a full version (download of the full version from the ESER homepage [1]). This causes text overlap between the two versions. We mention this to avoid a potential conflict with respect to self-plagiarism.

## Methods

The ESER Board instructed the former ESER President (S.W.) to divide the entire field of radiological polytrauma care into individual sections. S.W. assigned parts of the project to J.H. as part of his doctoral thesis at the Ludwig-Maximilian-University, Munich, Germany. Each section was processed and prepared according to a fixed schedule: determination of key issue(s), literature research, selection of literature, classification of literature, rating of literature, determining a level of evidence, suggesting a grade of recommendation, suggesting a statement for each key issue as a basis for the consensus conferences.

### Key issues

Each section was related to at least one key issue/question. The consensus conference members had to discuss and vote on one (or more) answer(s) to each key question, but were also allowed to delete or change answers.

### Literature research

For each key issue, a literature search was conducted with subjectively fitting keywords from the MeSH terms (Medical Subject Headings, [2]) including subjectively fitting synonymous keywords. The MeSH term search itself was performed using NCBI (National Center for Biotechnology Information) [2]. These keywords were used for searching through several databases: MEDLINE (via PubMed [3]), Cochrane Library [4] and Embase (via Ovid [5]). These databases were accessed via the Database Information System (DBIS) [6] of the University Library of the LMU Munich, where full text access was available for almost all journals. If there was only access to the abstract but not to the full text, the literature was excluded. Depending on the key issue the rate of such exclusion ranged between two and ten percent. Search terms and their connection were adapted to the individual databases. For guidelines, the databases of the NICE (National Institute for Health and Care Excellence [7]) and AWMF (Association of the Scientific Medical Societies in Germany [8]) were scanned. The AWMF database in German was included because S.W. and J.H. were able to understand it and, if necessary, translate it for the consensus conferences. The NICE search for guidelines was performed with the additional filter ‘Secondary Evidence’. The literature found was selected in a fixed order. The first step was to evaluate the relevance by title, then by abstract and, if necessary, by keyword search in the full text. Any literature not excluded in this first step was then subject to a more detailed second step examination of the inclusion and exclusion criteria on the basis of the full text. For more (very detailed) information about the literature search please refer to the guideline in full text [1].

### Inclusion/exclusion criteria for literature selection

The literature was selected on the basis of a catalogue of inclusion criteria. The search key words were determined together by S.W. and J.H. according to ‘sections’ and related ‘key issue(s)’. Data were excluded if at least one of the inclusion criteria listed below was not fulfilled.

1. Publication period from January, 1 2010 to February, 15 2019
2. Study population: n ≥ 50, adults (an age limit was not applied as children develop at different rates and therefore, from a radiological point of view, there may be a smooth transition to the body of an adult.)
3. Language of publication: English or German (German because S.W. and J.H. were able to understand it and, if necessary, translate it for the consensus conferences)
4. Full text accessible, free of charge via the university portal used
5. Clinical relevance of the literature included with regard to the key issue (subjective evaluation)
6. Additional criteria for guidelines:
  It is published as a guideline, i.e. using the word ‘guideline’ in the title
  The guideline is described as being current or no updated version is available
7. Additional criteria for studies:
  Allowed study types: meta-analyses, systematic reviews, randomised controlled trials, cohort studies, case–control studies, cross-sectional studies, before—after studies
  Outcome: *p*-value < 0.05 and/or confidence interval (CI) > 95%

### Classification, rating, and evidence level of studies

*Classification* In case of studies, the algorithm according to Hartling et al. [9] was used to classify the study type for included publications (e.g. prospective cohort study, case–control study, randomised controlled trial).

*Rating* Systematic reviews and meta-analyses were rated using AMSTAR 2 [10], randomised controlled trials using the Cochrane method [11], and cohort or case control studies using the Newcastle—Ottawa Scale (NOS) [11]. The Cochrane method and NOS have been performed according to the description in the Manual of Cochrane Germany and Association of Scientific Medical Societies in Germany (AWMF) [11] (e.g. were the items of PICO (population, intervention, comparator group and outcome) applied for a randomised controlled study? Were the sources of funding of the study published? [10]).

*Evidence Level* For every study, the level of evidence of was assigned using the scheme of the Oxford Centre for Evidence—Based Medicine in the 2011 version [12] (e.g. level 1 corresponds to systematic review of randomised trials or level 3 corresponds to a cohort study [12]).

### Grade of recommendation (GoR)

Using the evidence levels according to the AWMF Guidance Manual [13] one out of three possible Grades of Recommendation (GoR) was issued on each answer to a key question: A = ‘should/ should not’ with the meaning of ‘certainly should/ should not’; B = ‘ought/ ought not’ with the meaning of ‘probably/preferably should/should not’; 0 = ‘may be considered’ or ‘consider’. The GoR was based on the evidence level of the included studies: evidence level 1 led to a recommendation level ‘A’ (strong recommendation), evidence level 2 led to a recommendation level ‘B’ (recommendation) and evidence levels 3, 4, 5 lead to a recommendation ‘0’ (open recommendation). For statements from guidelines, the specific degree of recommendation was adopted from the guideline. Following the AWMF principle [14] and in case the used scale allowed us to do so, the consensus conference was able to increase or decrease the GoR by one degree of recommendation [14].

### Good clinical practice points (GPP)

If there was insufficient evidence in the literature included, the degree of recommendation—GPP (Good Clinical Practice Points, [15]-p. 27) was used. In contrast to GoR, GPP is based purely on the consensus of the experts. The GPP degrees of recommendation are identical to those for GoR: ‘A’ = strong recommendation, ‘B’ = recommendation and ‘0′ = open recommendation. For differentiation purposes, the degree of recommendation was therefore marked with GPP instead of GoR.

### Consensus development at the conferences

For each key issue, S.W. and J.H. proposed a statement with a corresponding level of recommendation (GoR or GPP) to the consensus conference. This served as a basis for the discussion during the consensus conference. If present in person, each member of the last and the current ESER Board (from 2017 until now) had exactly one equal vote. As the consensus conferences were during congress meetings, not all consensus members were able to present for the entire consensus conferences. According to our constitution, attendance by 2/3 of members was considered quorate for each vote on each statement. All members reviewed each statement during the publication process. The suggested statements and grades of recommendation as well as the corresponding literature were sent to the participants in advance by email.

The procedure for each key question is as follows (see also Table 1): For each section, the suggested statement(s) and GoR/GPP were presented by S.W. and a discussion was opened with the possibility of further questions, amendments, additions and objections. This stopped when there seemed to be a majority on the wording and the final suggested or amended statement was then voted on (with % consensus recorded). This was followed by a second vote on the GoR/GPP for this statement (again, the % consensus was recorded). If necessary, a new proposal for GoR/GPP was formed by discussion considering the rules for GoR/GPP (AWMF principle as described earlier) until at least simple majority was reached. Each voting was performed anonymously by holding a laser pointer within a given area, which was interpreted as ‘yes’, outside this area as ‘no’, and missing pointer signals as abstention (did not occur). All voting results were recorded (Tables 2, 3, 4, 5).

**Table 1:** Section 1: Polytrauma classification*

*Key question*: Which patients can be classed as polytrauma (and should therefore receive a whole-body computed tomography)?				
No	Statement(s)	Consensus *(positive votes on the statement, strength)*	Grade *(recommendation type, level)*	Consensus *(positive votes on the grade, strength)*
1.1	The assessment should be undertaken by the medical team in the Emergency Trauma Room** with regard to a potential life threatening situation and continuously reassessed with special regard to: Abnormalties of vital signs Injury mechanism Multiple body regions injuries and injury location Cofactors such as age, comorbidity, anticoagulant medication, pregnancy	100% strong	GPP A	100% strong
*Literature*: *abstracts* detected = 1697, excluded = 1662, full-text: rated = 35, excluded = 31, included = 4 (*evidence level of included literature* = guideline: [15]; level 2: [16–18])				
*Comments*: ESER does not assign a GoR because no evidence-based clear prospective definition was found in the literature. As a comment, ESER wants to recommend that the decision whether a patient is classed as polytrauma or not, should be taken by the trauma team leader in charge (a named person for each shift or patient). The trauma team leader has to decide in consultation with the rest of the trauma team, mainly the leading team members of Trauma Surgery, Anesthesiology and Radiology				

*In contrast to the following tables, Table 1 holds additional information for explanation in italics

**As there are several wordings for the room where polytrauma service is performed, ESER chose one of those terms and we decided to use ‘Emergency Trauma Room’ as wording in this Guideline. Common similar wordings are: Resuscitation Room or Shock Room

**Table 2:** Section 2: Structural points, key issue 1: CT location

*Key question*: Where should the CT-scanner be located with regard to a short service time and the lowest possible mortality rate of polytrauma patients?				
No	Statement(s)	Cons	Grade	Cons
2.1.1	The computer tomograph ought to be located in or directly next to the Emergency Trauma Room	71% weak	GoR B	100% strong
2.1.2	If this is not possible, the distance should not exceed 50 m	100% strong	GoR A	100% strong
2.1.3	The transportation route to further therapy (Interventional Radiology, Operating Room, Intensive Care/Therapy Unit, and in rare cases Coronary Unit) ought to be short	86% normal	GoR B	100% strong
*Literature*: detected = 367, excluded = 343, full-text: rated = 24, excluded = 18, included = 6 (guideline: [15, 19]; level 2: [20, 21]; level 3: [22, 23])				
*Comments*: A dual-room/ sliding gantry-CT may be considered in case of localisation in the Emergency Trauma Room

**Table 3:** Section 2: Structural points, key issue 2: CT type

*Key question*: Which computer tomography technology is needed for a polytrauma service?				
No	Statement(s)	Cons	Grade	Cons
2.2.1	Trauma Centres of the highest level of medical care should be equipped with a Multi-detector CT (MDCT) offering at least 64 simultaneous slices	100% strong	GoR A	86% normal
2.2.2	As isotropic scanning offers the advantages of high quality MPR (multiplanar reformations), a CT scanner ought to be preferred with at least 16 detector rows	86% normal	GPP B	86% normal
2.2.3	The computer tomographs ought to be equipped with current techniques for the reduction of radiation exposure, but this should not delay image reconstructions	100% strong	GoR B	86% normal
2.2.4	Dual-Energy/ Spectral imaging/ substraction imaging scanner may be considered	86% normal	GPP 0	71% weak
2.2.5	Trauma centres of the highest level of medical care should be technically equipped to a standard that will allow a perfusion CT of the brain	100% strong	GPP A	100% strong
2.2.6	Trauma centres of the highest level of medical care should be technically equipped to a standard that will allow a cardiac CT, if needed	14% none	-	-
*Literature*: detected = 615, excluded = 579, full-text: rated = 36, excluded = 28, included = 8 (guideline: [15, 24]; level 2: [25]; level 3: [26–30])				
Comments: As the technological development was fast in the last decade (the interval for literature inclusion), literature included reports on four row CT-scanners for polytrauma service. The consensus conference states them as obsolete

**Table 4:** Section 2: Structural points, key issue 3: Diagnostic Environment and Communication

*Key question*: Which work organization is recommended for polytrauma management with regard to workstation, data processing, image display and communication?				
No	Statement(s)	Cons	Grade	Cons
2.3.1	Depending on the individual framework conditions, each facility should enable the fastest possible initial image evaluation	100% strong	GoR A	100% strong
2.3.2	For this initial evaluation, an optimised workstation connected directly to the CT control console ought to be used	86% normal	GoR B	100% strong
2.3.3	These initial images should not exceed a maximum slice thickness of 5 mm	100% strong	GoR A	100% strong
2.3.4	Depending on the individual framework conditions, each institution should define a suitable infrastructure for the immediate oral as well as the further written exchange of information	100% strong	GoR A	100% strong
2.3.5	The transmission of findings may be considered to be supported with a selection of relevant images	86% normal	GoR 0	86% normal
2.3.6	There should be a way between hospitals to exchange CT images safely and timely	100% strong	GoR A	100% strong
*Literature*: detected = 850, excluded = 784, full-text: rated = 40, excluded = 31, included = 9 (guideline: [15, 19, 31–33]; level 3: [22, 34–36])				
*Comments*: Mobile devices may be useful in distributing relevant information

**Table 5:** Section 2: Structural points, key issue 4: Quality Management

*Key question*: What does suitable quality management entail for the radiological care of polytrauma patients?				
No	Statement(s)	Cons	Grade	Cons
2.4.1	Every radiological facility should establish targeted, individual quality management for the treatment of polytrauma	100% strong	GPP A	100% strong
2.4.2	Such quality management ought to define, monitor and continuously improve defined meaningful indicators	100% strong	GPP B	100% strong
2.4.3	Such a quality management ought to be integrated into and coordinated with a radiological as well as a clinical overall quality management	86% normal	GPP B	86% normal
*Literature*: No literature search was conducted				
*Comments*: Quality management has long been established in industry and is increasingly proving itself in medical applications. Quality management is desirable, but so far little suitable reliable information is available. More precise recommendation on quality management should be the subject of future research and also of radiological or clinical consensus conferences. As a first choice useful parameters may be: time-to CT-service; time of CT-service; time-to therapy; total dose; image quality; errors in first, second and third readings; number and frequency of morbidity and mortality conferences				

An agreement of voting was achieved by a consensus strength of more than 50% of the present votes.

The consensus strength was graduated according to the AWMF rules ([14]-p.40) as follows: Strong (strong agreement): > 95% of votes; Normal (normal agreement): > 75–95%; Weak (majority agreement): > 50–75% and None (no agreement): < 50% (Tables 6, 7, 8, 9, 10).

**Table 6:** Section 3: Extended Focused Assessment with Sonography for Trauma (eFAST)

*Key question*: What significance does the eFAST examination have in the Emergency Trauma Room treatment of polytrauma patients?				
No	Statement(s)	Cons	Grade	Cons
3.1	eFAST should be used as part of the Primary Survey	100% strong	GoR A	100% strong
3.2	eFAST should be implemented simultaneously with other measures, i.e. without additional expenditure of time for the overall care. If this is not possible, eFAST should not delay CT	100% strong	GoR A	100% strong
*Literature*: detected = 699, excluded = 681, full-text: rated = 18, excluded = 6, included = 12 (guideline: [15, 19, 37–39]; level 1: [40]; level 2: [41, 42]; level 3: [43, 44]; level 4: [45]; level 5: [46])				
*Comments*: eFAST ought to be a screening for diagnostic findings requiring immediate treatment. With this meaning eFAST is a filter to (maybe temporarily) exclude (very few) patients from CT-scanning because of reasons where the time effort of CT is expected to lead to higher mortality. Such findings in unstable patients may be tension pneumothorax, pericardial tamponade, massive bleeding in the pleural or peritoneal spaces

**Table 7:** Section 4: Conventional Radiography

*Key question*: What is the significance of conventional X-rays and under what conditions are conventional X-rays preferred to computer tomography in the Emergency Trauma Room treatment of polytrauma patients?				
No	Statement(s)	Cons	Grade	Cons
4.1	For the clarification of polytrauma, CT should be preferred to X-ray	100% strong	GoR A	100% strong
4.2	In addition to an eFAST, conventional X-ray should also be immediately available	100% strong	GoR A	100% strong
*Literature*: detected = 893, excluded = 845, full-text: rated = 18, excluded = 7, included = 11 (guideline: [15, 19, 24, 47, 48]; level 2: [49, 50]; level 3: [51–53]; level 5: [46])				
*Comments*: None

**Table 8:** Section 5: Whole Body CT – Positioning, key issue 1: patient orientation

*Key question*: How does head- or feet-first positioning affect a polytrauma – WBCT scan?				
No	Statement(s)	Cons	Grade	Cons
5.1.1	If it is logistically possible, the patient ought to be positioned on the examination table with her/his feet in front of the gantry	86% normal	GPP B	86% normal
5.1.2	Otherwise, the scan ought to be done head first	100% strong	GPP B	86% normal
Literature: detected = 328, excluded = 323, full-text: rated = 5, excluded = 5, included = 0				
Comments: Although without any evidence, the advantages of feet-first positioning appear to be clear in terms of reduced radiation exposure of personnel, reduced artifacts due to cable routing, reduced cable routing problems, easier accessibility to the head

**Table 9:** Section 5: Whole Body CT – Positioning, key issue 2: Arm position

Key question: How do different arm positions of patients with polytrauma impact computed tomography scans with respect to radiation exposure, image quality and scan duration?				
No	Statement(s)	Cons	Grade	Cons
5.2.1	Depending on the patient or their clinical condition, the arms should be positioned down (time-optimised) or up (dose-optimised)	86% normal	GoR A	100% strong
5.2.2	For a time-optimised protocol (e.g. in haemodynamically unstable patients), arms ought to be crossed over the trunk in such a way that the hardening artifacts are distributed to best effect over the z-axis (time-optimised procedure equals quick)	100% strong	GoR B	100% strong
5.2.3	For a dose-optimised protocol (prerequisite: haemodynamically stable patients), arms for the CT scan of the trunk ought to be positioned above the head unless there is evidence of a significant injury to the corresponding local shoulder region (dose-optimised procedure equals lower radiation)	86% normal	GoR B	100% strong
*Literature*: detected = 695, excluded = 673, full-text: rated = 22, excluded = 16, included = 6 (guideline: [15]; level 2: [54–56]; level 3: [57, 58])				
*Comments*: The positioning of the arms above the head costs time as well as coming with further drawbacks, however it does reduce the dose for the trunk. The positioning with crossed forearms over the abdomen distributes the hardening artifacts over the abdomen, is very fast and risk-free, easy to fix and favours the outflow of the given intravenous contrast media. In addition, the entire upper limb, which is often injured, is often imaged in this way

**Table 10:** Section 6: Whole Body CT – Protocol, key issue 1: CT scout

*Key question*: What diagnostic value does the scout of a whole-body CT scan have in the case of a polytrauma patient and how should it be prepared?				
No	Statement(s)	Cons	Grade	Cons
6.1.1	The scout(s) ought to represent the entire body	100% strong	GoR B	100% strong
6.1.2	For a dose-optimised protocol, separate topograms should be prepared for the cranial CT (at least lateral projection) and the rest of the body (at least anterior—posterior projection). If the arms are raised, this should be done before the body topogram is prepared	100% strong	GPP A	86% normal
*Literature*: detected = 1195, excluded = 1168, full-text: rated = 27, excluded = 16, included = 11 (guideline: [19, 37, 59, 60]; level 2: [55]; level 3: [27, 29, 57, 61–63])				
*Comments*: The CT scout does not only hold information of important findings, it also is the basis to calculate the dose modulation during the CT scan. For protocols with elevated arms, a dose reduction only affects cases where the arms were raised before the CT scout was performed				

## Results

The results of the consensus conferences are presented here and structured into ten sections. Each section may be subdivided into several key issues that were presented as tables in the following. Each table also holds a collection of ‘key literature’ that corresponds to the literature included. The tables also include a path through the literature classification as well as the evidence levels of the included literature (Tables 11, 12, 13, 14, 15).

**Table 11:** Section 6: Whole Body CT – Protocol, key issue 2: Cranial CT

Is an unenhanced cranial scan preferred to a cranial scan with contrast medium as first imaging option in the whole-body tomography scan of the polytrauma patient?					
No	Statement(s)	Cons	Grade	Cons	
6.2.1	The full body tomography scan of the polytrauma patient should begin with an unenhanced cranial CT scan	100% strong	GoR A		100% strong
6.2.2	Depending on the findings and symptoms, an additional cranial CTA (computed tomography angiography) may be considered as useful	86% normal	GoR 0	86% normal	
*Literature*: detected = 2266, excluded = 2228, full-text: rated = 38, excluded = 13, included = 25 (guideline: [15, 19, 64]; level 2: [25, 28, 55, 56, 65]; level 3: [16, 26, 27, 29, 51, 58, 61, 66–75])					
*Comments*: Virtual unenhanced CT imaging with Dual Energy techniques should undergo more scientific evaluation. Maybe this method will allow single enhanced cranial CT scanning with sufficient detection rates of intracranial bleedings by virtual unenhanced imaging. If so, this may have the potential for both speeding up service and reducing the dose

**Table 12:** Section 6: Whole Body CT – Protocol, key issue 3: Cervical Neck/Spine

How should the head/neck region in the standard whole-body tomography protocol be performed in a polytrauma patient with regard to contrast agent administration and image calculation?				
No	Statement(s)	Cons	Grade	Cons
6.3.1	With a protocol that is not dose-optimised, the neck region should be included in the whole body tomography scan with intravenous contrast medium in such a way that the neck arteries and brain base arteries are well opacified	100% strong	GoR A	100% strong
6.3.2	If only a bony injury is suspected in the cervical spine, the scan may be considered without the administration of contrast medium within the framework of a dose-optimised protocol	71% weak	GoR 0	71% weak
6.3.3	For dose reasons, the cranial scan ought not to be extended to the cervical spine	86% normal	GPP B	86% normal
6.3.4	Axial image reconstruction should be performed in thin slices with both a soft tissue and a bone kernel	100% strong	GoR A	100% strong
6.3.5	Image reformation should take place at all three orthogonal standard planes	100% strong	GoR A	86% normal
6.3.6	The neck may be considered as part of the body scan as long as a second image reconstruction with a Field-of-View adapted to the neck is performed	100% strong	GoR 0	100% strong
*Literature*: detected = 3557, excluded = 3507, full-text: rated = 50, excluded = 16, included = 34 (guideline: [15, 19, 47, 76–84]; level 1: [85] level 2: [25, 28, 55, 56, 65, 75, 86]; level 3: [16, 27, 29, 51, 61, 66, 68, 70, 72–74, 87–89])				
*Comments*: None

**Table 13:** Section 6: Whole Body CT – Protocol, key issue 4: contrast phase

*Key question*: What is the optimal phase for contrast enhanced emergency polytrauma imaging?				
No	Statement(s)	Cons	Grade	Cons
6.4.1	The choice of the injection protocol should be individually adapted to the patient and their clinical condition, in particular with regard to dose aspects and required diagnostic significance	86% normal	GPP A	86% normal
6.4.2	An unenhanced phase may be considered to be performed in case of question of blood components outside a vascular lumen	57% weak	GoR 0	57% weak
6.4.3	For a given indication, it may be considered to calculate an unenhanced phase using the dual-energy technique	100% strong	GoR 0	100% strong
6.4.4	Purely unenhanced CT imaging should not be performed on the trunk of the body	100% strong	GoR A	86% normal
6.4.5	A split bolus protocol ought to be part of a dose-optimised protocol	71% weak	GPP B	57% weak
6.4.6	Where a split bolus protocol identifies questionable relevant findings, the region in question ought to be supplemented with an additional appropriate further phase	100% strong	GPP B	100% strong
6.4.7	For a protocol with a focus on highest diagnostic precision, at least the upper abdomen should be depicted in both the arterial and venous phases	86% normal	GoR A	100% strong
6.4.8	For image findings suspicious of active bleeding, at least two temporally separated contrast phases ought to be present to estimate the activity	100% strong	GoR B	86% normal
*Literature*: detected = 2518, excluded = 2450, full-text: rated = 68, excluded = 22, included = 46 (guideline: [15, 19, 24, 48, 79, 90–98]; level 1: [99]; level 2: [25, 26, 28, 55, 56, 65, 89, 100–103]; level 3: [16, 27, 29, 51, 52, 54, 57, 58, 61–63, 70, 72, 74, 75, 104–108])				
*Comments*: The section deals with intravenous contrast media. Mainly for time reasons oral or rectal filling is inappropriate / obsolete

**Table 14:** Section 6: Whole Body CT – Protocol, key issue 5: Injection of Contrast Media

*Key question*: What do the WBCT protocol parameters manifest itself in case of a polytrauma patient regarding the application of contrast medium?				
No	Statement(s)	Cons	Grade	Cons
6.5.1	For a split bolus, the larger component ought to be used for the first injection (portal-venous phase part)	100% strong	GoR B	100% strong
6.5.2	A saline flush should be used at the end of each contrast medium injection	100% strong	GoR A	100% strong
6.5.3	Each facility ought to maintain multiple standard injection protocols and consider individual patient characteristics for injection	86% normal	GPP B	86% normal
6.5.4	Each institution should critically and regularly check the resulting image quality, inspect the protocols regarding this and a possible reduction of the contrast medium quantity	100% strong	GPP A	86% normal
*Literature*: detected = 3111, excluded = 3059, full-text: rated = 52, excluded = 25, included = 27 (guideline: [19, 79, 109, 110]; level 2: [28, 55, 56, 111]; level 3: [26, 27, 29, 51, 54, 58, 61, 63, 70, 72, 74, 75, 89, 101–103, 105, 108, 112])				
*Comments*: The contrast medium injection protocols are quite inconsistent. The Sections 6.4 and 6.5 overlap and should be merged in upcoming guideline updates

**Table 15:** Section 7: Whole Body CT – Special protocols, key issue 1: CT—urography

Key question: What are the indications for extended imaging of the urinary tract?				
No	Statement(s)	Cons	Grade	Cons
7.1.1	The indications should be taken in conjunction with the guideline from the European Society of Urogenital Radiology (ESUR)	100% strong	GPP A	86% normal
7.1.2	A urographic phase should not delay other immediately necessary life-sustaining therapy	100% strong	GPP A	100% strong
7.1.3	If necessary, a urographic phase may be considered up to a few hours after the initial CT without further injection of contrast media	100% strong	GPP 0	100% strong
7.1.4	If in situ, a bladder catheter should be clamped first before performing the urographic phase	100% strong	GPP A	100% strong
7.1.5	In case of unclear findings of the bladder and urethra, an additional retrograde filling may be considered	100% strong	GoR 0	100% strong
*Literature*: detected = 2639, excluded = 2615, full-text: rated = 24, excluded = 15, included = 9 (guideline: [15, 19, 113–118]; level 3: [100])				
*Comments*: None				

## Discussion and conclusions

For a detailed literature discussion of more than 50 print pages we have to refer to the guideline in full length (access via ESER homepage [1]).

As a relatively young society, ESER overcame challenges during the guideline development, consensus and publication process. The members of the consensus group were distributed throughout diverse nations, making the necessary distribution of information and communication time consuming. Financial limitations restrict the whole group from coming together face to face to only once or twice a year, during the European congresses of radiology in Vienna and the ESER congress meetings. This huge project required two sittings of the consensus group to adequately provide time for discussion, this, in addition to restrictions of the SARS-Cov-2 situation and cancellation of the European Congress of Radiology (ECR) 2020 caused unexpected delay in manuscript production (Tables 16, 17, 18, 19).

**Table 16:** Section 7: Whole Body CT – Special protocols, key issue 2: CT—angiography

*Key question*: Under which conditions should the standard WBCT protocol of the polytrauma patient be adapted with regard to CT-angiography of the extremities, aorta or intestinal/mesenteric?				
No	Statement(s)	Cons	Grade	Cons
7.2.1	CTA of the extremities ought not to be a standard part of the whole body CT polytrauma protocols	100% strong	GPP B	100% strong
7.2.2	In the case of an extension of the whole body CT scan, identified prior to the examination, the guidelines of the respective radiological -subspeciality societies should be taken into account, e.g. cardiovascular, abdominal	100% strong	GPP A	86% normal
*Literature*: detected = 3464, excluded = 3408, full-text: rated = 56, excluded = 20 included = 36 (guideline: [15, 19, 79, 91, 92, 94, 96–98, 119–132]; level 1: [99, 133]; level 3: [16, 52, 63, 72, 103–105, 134–137])				
*Comments*: None

**Table 17:** Section 8: Whole Body CT – Reading/ Reporting

*Key question*: What is the procedure for the assessment and evaluation of the whole body tomography scan in the case of a polytrauma patient to be as quick and accurate as possible?				
No	Statement(s)	Cons	Grade	Cons
8.1	The entire initial WBCT should be evaluated three times (primary, secondary, tertiary) for a very high level of diagnostic safety	100% strong	GoR A	100% strong
8.2	In total, reading should be carried out by at least two different radiologists, at least one of whom should be board certified. In each case, the assessment should be based on the ABCDE scheme	100% strong	GPP A	100% strong
8.3	Scout assessment: The scout should be interpreted immediately in order to triage the patient and/or adapt the scan protocols as required	57% weak	GPP A	57% weak
8.4	Primary assessment: As soon as the first CT series are available they should be evaluated immediately with the focus on acutely relevant findings (ABCDE scheme)	100% strong	GPP A	86% normal
8.5	Primary documentation and communication: should happen immediately verbally and be handled adequately according to the institutional setting and should be documented	100% strong	GPP A	86% normal
8.6	Secondary assessment: should also be carried out as quickly as possible, but at least within one hour after the primary assessment and based on the final images. Any relevant changes to the primary assessment should be communicated immediately and be documented	100% strong	GPP A	100% strong
8.7	Tertiary assessment: Should take place within 24 h at latest. In case of relevant changes in findings, these should also be communicated immediately and any changes in findings should be documented. In cases where the second report was authorised by a Board certified Radiologist, this should be done as an addendum	100% strong	GPP A	100% strong
*Literature*: detected = 2241, excluded = 2193, full-text: rated = 48, excluded = 31, included = 17 (guideline: [15, 19, 31, 138–140]; level 2: [18]; level 3: [62, 66, 141–146]; level 4: [147, 148])				
*Comments*: Reading polytrauma CT three times may seem time-consuming. The consensus group interpreted the first reading as the reading of the very first images (e.g. 1 mm axial slices in soft tissue kernel with MPR views from these data as provided automatically with first, often oral report. This includes reading of the scout but is not limited to the scout). The second reading means the reading of the final reconstructed images as stored in PACS (picture archiving and communication system) with written report. In most cases, the first and second reading will be performed by the same radiologist. Finally, the third reading should be done by a different radiologist. For CT scans during regular working hours this may be the reading performed by an attending radiologist (maybe in parallel with the second reading together with the radiologist who did the first reading). For CT scans during on call periods, the third reading may be performed in the morning of the next day. This may be the Radiologist on the next routine in-hours shift or next on-call Radiologist. As some European countries offer Emergency Radiology as a certified radiological subspecialty and some do not, ESER offers a European Diploma in Emergency Radiology as an international qualification. Although desirable, ESER does not mandate such a formal national or international Emergency Radiology qualification. Instead, ESER emphasises that in each case at least the second or the third reading has to be performed by a board certified radiologist with fundamental experience in Emergency Radiology

**Table 18:** Section 9: Interventional Radiology

*Key question*: In which cases should interventional radiology be consulted?				
No	Statement(s)	Cons	Grade	Cons
9.1	The indications should be taken in conjunction with the guideline from the relevant radiological subspecialty societies CIRSE (Cardiovascular and Interventional Radiological Society of Europe) and ESNR (European Society of Neuroradiology)	100% strong	GPP A	75% weak
9.2	Interventional (neuro-) radiology should be available 24/7 for consultation and treatment within a locally agreed timely manner	100% strong	GPP A	100% strong
*Literature*: None				
*Comments*: None

**Table 19:** Section 10: Summary: A proposal for two WBCT—Protocols in the Trauma Care

*Key question*: Is one standard CT protocol sufficient?				
No	Statement(s)	Cons	Grade	Cons
10.1	Within the framework of radiological polytrauma management, at least two different WBCT protocols should be maintained as institutional standards. One should be optimised with regard to radiation dose yielding high diagnostic validity but prioritising lower radiation burden (Dose Protocol). The other one is a compromise, prioritising rapid diagnosis and very high diagnostic validity over the potential risks of increased radiation burden (Time/Precision Protocol)	100% strong	GPP A	100% strong
10.2	The Time/Precision Protocol should be preferred for polytrauma patients with life-threatening injuries or haemodynamically unstable conditions	88% normal	GPP A	100% strong
10.3	The Dose Protocol should be preferred for polytrauma patients provided they do not have obvious life-threatening injuries or are haemodynamically unstable	100% strong	GPP A	100% strong
*Literature*: No literature search was conducted				
*Comments*: It has been proven that the maintenance of a protocol standard for whole-body CT after polytrauma increases the probability of survival [149]. As a possible consequence of this fact, the experts at the conference observed an increase in Emergency Trauma Room admissions who subsequently receive a WBCT. In parallel, the ESER experts share the impression that the number of patients with minor injuries who undergo WBCT has also increased. The consensus group concluded that a single standard protocol can rarely do justice to this varied situation. A more refined but nevertheless simple differentiation would be desirable with regard to the essential influencing parameters: Injury severity, patient condition, patient age including the probability of relevant comorbidities and/or medication, dose aspects especially with regard to patient age. The other previous recommendations remain unaffected				

## Limitations

- Only one person was involved in suggesting key issues (S.W.)
- Literature search was limited to two persons (S.W., J.H.)
- Literature preparation (exclusion, inclusion, grading) was also limited to S.W., J.H.
- The preparation of the consensus conference(s) including suggested statements and respective grading of them was limited to S.W., J.H.
- Literature inclusion was limited to free full access via the Ludwig-Maximilians University of Munich, Germany. However, this quote was about 95 percent in mean and always above 90 percent.
- German was the only non-English language that could be included in the literature search (because S.W. and J.H. were able to understand and translate it for the consensus conference members).
- The guideline does not cover special topics like paediatric patients or interventional radiology; these are an aspiration for future editions

## Conclusions

By developing this guideline, the ESER aimed to redress the lack of dedicated separate independent radiological recommendations for radiological polytrauma service. ESER recommends that a patient should first be assessed as ‘polytrauma’, who will therefore receive whole-body CT (WBCT) or ‘non-polytrauma’ (assess patient as a ‘normal’ emergency patient in the Emergency Trauma Room: do not automatically perform a WBCT). For a polytrauma service, the CT distance to the Emergency Trauma Room should not exceed 50 m—the closer, the better [15, 19, 20]. The CT used should offer 64 rows and modern technology (cardiac capability is welcome but not essential) [15, 24, 26–29]. Radiology departments as part of Trauma centres should optimise communication and drive quality assurance/ management [15, 31, 32, 34, 150, 151]. eFAST should be part of the primary survey and Radiography should be immediately available [15, 37–41, 43, 46]. ESER prefers to position patients ‘feet first’. In case of stable patients and if possible, arms should be elevated for dose reduction (only if this is done prior to the body scout) [15, 54, 56, 57]. CT scouts preferably should present the whole patient (but may consist of different parts) [37, 63], may replace chest radiographs [15, 51] and sometimes also provide justification for deviating from the standard protocol by choosing different contrast phases or extend scanning to other suspicious body regions. The unenhanced cranial CT scan certainly should be done first [19]. At least when using the ‘dose’ protocol (WBCT variant B), the cranial CT scan should only cover the brain. For unstable patients, the midface/neck/cervical spine should be scanned together with the chest using arterial contrast including the arteries of the skull base [70] (for stable patients a separate low-dose scan with or without contrast enhancement may be an alternative before lifting the arms). A split bolus protocol should probably be used with a dose-optimised protocol [74, 101, 108]. Otherwise, ESER recommends overlapping scans of the neck/chest/upper abdomen in arterial phase and the abdomen/pelvis in portal-venous phase [26, 98, 99]. For specific questions related to the urogenital, interventional, (cardio)vascular or paediatric specialties, ESER recommends using existing guidelines from the respective (sub)societies [117, 118, 129–131]. First images should be available, read and communicated as fast as possible using the ABCDE approach [19, 66, 143]. In the second step ‘perfect’ images should be calculated (in both soft and enhancing kernels) and be interpreted (and archived) at least in the three standard planes, respectively [19, 65, 66, 144]. Interpretation should occur three times (immediately using first images, immediately reassessed using the final images and reassessed again by a different radiologist within 24 h) [141, 144, 146].

ESER endorses abandoning a ‘one-size-fits-all-concept’ ([63]-p.1142). Instead, ESER recommends introducing a double-track whole-body tomography protocol concept with a ‘Dose Protocol’ and a ‘Time/Precision Protocol’. Obviously, the choice between the two variants should be based on the individual clinical presentation and vital parameters of the polytrauma patient. The ‘Dose protocol’ should be designed in such a way that the patient is exposed to the lowest possible radiation exposure despite sufficient image quality in order to ensure a reliable diagnosis of injuries (often young and stable patients with dramatic injury history and a Glasgow Coma Scale = 15). A dose far below 20 mSv should be aimed for. A good potential ‘Dose protocol’ may consist of an unenhanced head scan, low dose CT of the midface/ neck/ cervical spine (with or without contrast enhancement), elevation of the arms, scout of the trunk, and a single pass scan of chest/ abdomen and pelvis using a split bolus injection protocol with a resulting arterial/venous mixed contrast of all vessels and organs. In (few) cases where a ‘Dose protocol’ scan leaves potentially important findings unclear, another CT scan should be performed accordingly.

**Fig. 1:**
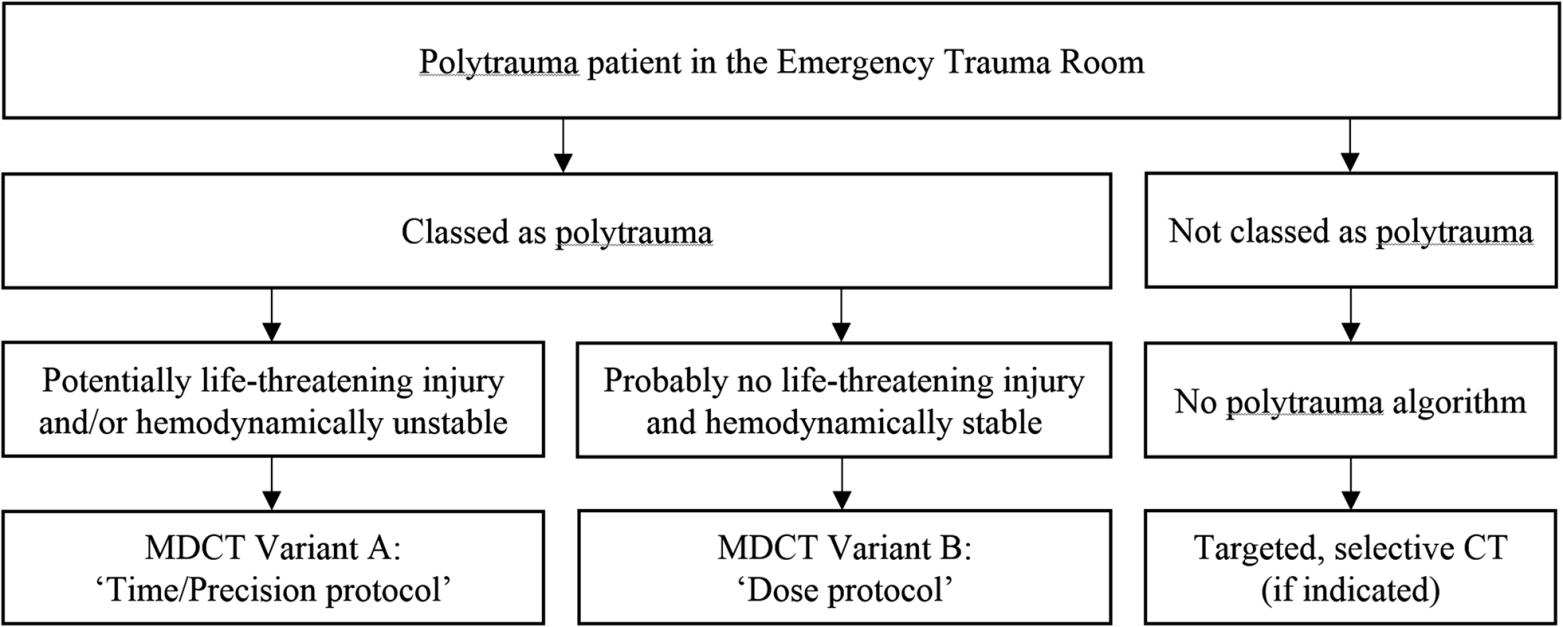
Decision guidance for polytrauma CT imaging. First, a potential polytrauma patient should be re-evaluated in the Emergency Trauma Room whether the criteria for a classification as polytrauma (Table 1) is given. If so, and in the case of a severe clinical presentation with life-threatening injuries and/or haemodynamic instability, the polytrauma ‘Time/Precision protocol’ (whole-body CT (WBCT) variant A) is applied. If the patient is also classed as polytrauma but does not fulfil criteria for MDCT protocol variant A, the ‘Dose protocol’ (WBCT variant B) may be used. Otherwise, the patient should receive imaging like other emergency patients

In contrast, the ‘Time/Precision protocol’ is optimised for very fast, very high diagnostic accuracy and will more or less correspond to the institutional protocol used so far. The key advantage is the more sensitive detection of active bleeding [15, 19, 107, 108]. The assignment of the polytrauma patient to one of the two protocols is shown in Fig. 1.

The ESER hopes that this guideline motivates diverse national and international radiological societies to come together in order to refine the statements over time. The ESER acknowledges that these guidelines do not focus on the radiological polytrauma service for children and Interventional Radiology. Rather the ESER invites the corresponding national and international radiological (sub)societies to contribute in the future. Where the guidelines do overlap with other radiological communities on topics such as Musculoskeletal, Abdominal & Urogenital imaging, the ESER anticipates arriving at a consensus in the future.

ESER sees this as way to gain visibility for national societies in the field and in particular to strengthen the role of Radiology in upcoming interdisciplinary polytrauma guideline processes. As ESER is active in the whole field of emergency radiology, we also aim to expand the guideline to non-traumatic Emergency Imaging in upcoming versions.

## Data Availability

Data sharing is not applicable to this article as no datasets were generated or analysed during the current study. Included literature was however searched as described in databases that are publically available. For more detailed information about the literature search and inclusion process you may refer to the full ESER Guideline that will be published at the ESER homepage (www.eser-society.org).

## Abbreviations

AWMF: Association of Scientific Medical Societies in Germany
CI: Confidence interval
CIRSE: Cardiovascular and Interventional Radiological Society of Europe
CT: Computed tomography
CTA: Computed tomography angiography
DBIS: Database information system
ECR: European Society of Radiology
EDiR: European diploma in radiology
eFAST: Extended focused assessment with sonography for trauma
ESER: European Society of Emergency Radiology
ESNR: European Society of Neuroradiology
GoR: Grade of recommendation
GPP: Good clinical practice points
MDCT: Multi-detector computed tomography
MeSH: Medical subject headings
MPR: Multiplanar reformation
NCBI: National Center for Biotechnology Information
NICE: National Institute for Health and Care Excellence
NOS: Newcastle–Ottawa Scale
PACS: Picture archiving and communication system
PICO: Population, intervention, comparator group and outcome
WBCT: Whole-body computed tomography
